# Characteristic Variabilities of Subnanometer EOT La_2_O_3_ Gate Dielectric Film of Nano CMOS Devices

**DOI:** 10.3390/nano11082118

**Published:** 2021-08-20

**Authors:** Hei Wong, Jieqiong Zhang, Hiroshi Iwai, Kuniyuki Kakushima

**Affiliations:** 1 Department of Electrical Engineering, City University of Hong Kong, Hong Kong, China; jieqzhang3-c@my.cityu.edu.hk; 2 Frontier Research Center, Tokyo Institute of Technology, Yokohama 226-8501, Japan; iwai.h.aa@m.titech.ac.jp (H.I.); kakushima.k.aa@m.titech.ac.jp (K.K.)

**Keywords:** surface roughness, ultrathin film, nano CMOS, variability

## Abstract

As CMOS devices are scaled down to a nanoscale range, characteristic variability has become a critical issue for yield and performance control of gigascale integrated circuit manufacturing. Nanoscale in size, few monolayers thick, and less thermally stable high-k interfaces all together cause more significant surface roughness-induced local electric field fluctuation and thus leads to a large device characteristic variability. This paper presents a comprehensive study and detailed discussion on the gate leakage variabilities of nanoscale devices corresponding to the surface roughness effects. By taking the W/La_2_O_3_/Si structure as an example, capacitance and leakage current variabilities were found to increase pronouncedly for samples even with a very low-temperature thermal annealing at 300 °C. These results can be explained consistently with the increase in surface roughness as a result of local oxidation at the La_2_O_3_/Si interface and the interface reactions at the W/La_2_O_3_ interface. The surface roughness effects are expected to be severe in future generations’ devices with even thinner gate dielectric film and smaller size of the devices.

## 1. Introduction

The continual downsizing process of CMOS devices has been slowed down in recent years due to difficulties encountered when approaching both physical and technological limits, which are believed to be of a couple of nanometers in feature size [1,2]. The technological limits can be split into two different categories. One group refers to the minimum achievable dimensions such as the fin width in a FinFET structure or the diameter of a silicon nanowire in a gate-all-around (GAA) structure. These limits are not only governed primarily by the available lithography techniques, but also by the gate dielectric thickness in the sense of equivalent oxide thickness (EOT), which is limited by the thin layer deposition techniques and by some other processing steps that may result in the EOT degradation [2]. However, as device gate length being scaled to a few nanometers and the gate dielectric thickness approaching the atomic scale, it is expected that the thickness fluctuation of the gate dielectric, the surface roughness on the fins in the FinFET structure or on the nanowire in the Gate-All-Around (GAA) structure, the gate dielectric/silicon interface and the gate dielectric/metal gate interface could become comparable to the gate dielectric thickness itself [3,4]. They are basically non-scalable, and it results in the second group of technological limits which are the process and device variabilities, and it becomes the most critical issue for the yield and performance control of gigascale integrated circuit manufacturing [3,4,5].

Thanks to the introduction of high-k materials, the physical thickness of the gate dielectric is now still maintained well above the atomic scale for the current nano CMOS technology [2]. When the EOT gate dielectric is further scaled down to a half nanometer range, the surface roughness fluctuation of this ultrathin film will become a serious issue and particular cares need to be taken in the process design and the device variability control [4,5]. This issue would become more severe because of further EOT scaling and the worsened interface by the post high-k processing steps. Although the state-of-the-art thin film deposition technology is able to control the thickness variation down to the atomic scale, the heterogeneous and less thermal stable natures of high-k material often make the silicon/high-k interface and metal gate/high-k interface much rougher than a thermal growth Si/SiO_2_ interface [3]. It is well-known that one of the main causes of channel mobility degradation in the MOS structure is mainly due to the surface scattering of the rough interface. The MOS technology development has experienced continual mobility degradation along with device downsizing for decades [1]. The gate leakage current is now the second technological issue arising from the surface roughness [4,5].

## 2. Experimental Details

In this work, the La_2_O_3_ MOS capacitors were fabricated with the local oxidation of silicon (LOCOS) isolation structure. After the standard RCA cleaning processing, a 200 nm thick silicon oxide layer was thermally grown. The active device regions were defined with photolithograph and buffered hydrogen fluoric (HF) acid etching. Just before the sample being loaded into the multi-chamber high-k/metal gate deposition system, the wafer was further etched with a diluted HF solution for 5 min to remove the native chemical oxide. The La_2_O_3_ film with 5 nm thick was deposited by e-beam evaporation in an ultrahigh vacuum chamber. The sample was then transported within the multi-chamber system with the help of a robot arm to the magnetron sputtering chamber for tungsten film deposition. A tungsten layer of 3 nm thick was deposited which was further covered with a 50 nm thick oxygen-free TiN layer for best protection of the La_2_O_3_ film from moisture. The hygroscopic nature of La_2_O_3_ does not only significantly degrade its insulating property, but it also results in a significant increase in the surface roughness [6,7,8,9]. During high-k/metal gate processing steps, the wafers were not exposed to the ambient or had any direct contact with the operator. The possibility of contamination should have been greatly minimized. The electrodes were then patterned with a lithography process. The TEM pictures were taken with JEOL JEM-2011F with 200 kV and *C*s = 0.5 mm. For electrical measurements, the samples were placed in a semi-automatic wafer probe with light and electromagnetic shielding. The current-voltage characteristics were measured with an Agilent B1500A Semiconductor Device Parameter Analyzer and the capacitance-voltage measurement was performed with an Agilent 4284A precision LCR meter.

## 3. Results and Discussion

### 3.1. Effects of Thermal Annealing on Interface Structure and Capacitance Value

**Figure 1:**
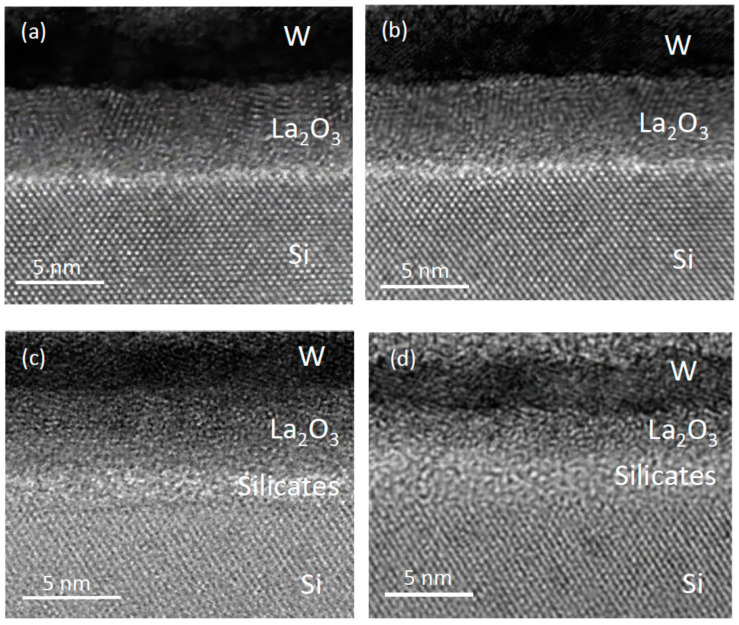
Transmission electron microscopy (TEM) picture of (**a**) as-deposited La_2_O_3_ film; (**b**) with 300 °C annealed, (**c**) 500 °C annealed and (**d**) 600 °C annealed sample. Samples with 500 °C and 600 °C annealing show significant growth in interface roughness and interface silicate layer.

Figure 1 shows some typical TEM pictures for as-deposited W/La_2_O_3_/Si stack and stacks subjected to different temperatures annealing for 30 min. The surface roughness of high-k gate dielectrics is governed by the processes involved before, during, and after the thin film deposition. In high-k/metal gate fabrication, for the state-of-the-art CMOS technology, excellent surface flatness can be obtained by using the ALD technique and the in situ metal film deposition [3]. However, it is difficult to maintain this near ideal surface. High-k metal oxides are more ionic and are less stable on silicon and in interaction with a gate electrode. Depending on the processing temperature, on the partial pressure of oxygen, several different chemical reactions and physical modifications (such as local crystallization) may take place at both interfaces and even in the bulk of the high-k layer [6,7,8,9,10]. Thermal annealing is thus an excellent processing step to testify the stability of the high-k film and its interfaces and to probe with the optimal process steps for device fabrication. In Figure 1a, it is noted for an as-deposited film that the La_2_O_3_/Si interface is sharp and it is also quite smooth. An earlier study based on the same fabrication process and parameters found that the interface roughness is 0.08 nm. The data were obtained based on Gaussian interface edge estimation on an aberration-corrected high-resolution TEM picture [9]. For the sample that underwent 300 °C annealing (see Figure 1b), no notable interface growth was found. However, it could still be observed at some spots that the interface becomes rougher. The typical interface roughness value after 300 °C annealing is about 0.12 nm [9]. The rougher interface after 300 °C annealing can be attributed to the local oxidation of the silicon substrate [10]. For samples with annealing temperatures of 500 °C and 600 °C (see Figure 1c,d), significant growth of the interfacial silicate layer was found. When analyzed with aberration-corrected high-resolution TEM, it was observed rough surfaces at La_2_O_3_/Si interfaces after the thermal treatment at 300 °C. This change was attributed to the phase separation effects [9]. However, this observation can be also correlated to the local interface oxidation due to the trace amount of oxygen diffused from the tungsten electrode [3]. Wong later attributed this observation to the local interface oxidation due to the trace amount of oxygen diffused from the tungsten electrode [10]. The amorphous silicate layer formed by the oxygen diffusion, which is rather non-uniform, can make the interface rougher. Both W/La_2_O_3_ and La_2_O_3_/Si are much rougher because of non-uniform interface reactions for samples with thermal annealing. The low-k silicate layer governed the lowest achievable EOT. Hence, 300 °C annealing should be the higher annealing temperature for the La_2_O_3_/Si stack from the EOT point of view.

**Figure 2:**
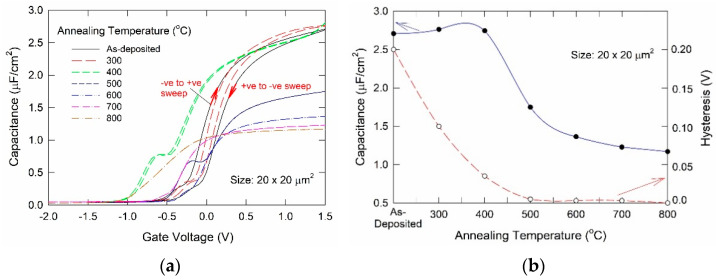
(**a**) Bi-directional high-frequency capacitance-voltage measurements on W/La_2_O_3_/Si capacitors with different annealing temperatures after the La_2_O_3_ deposition; (**b**) plot of maximum capacitance and hysteresis of the C-V characteristics as a function of annealing temperature.

To investigate the effective thickness and trap level of the samples, we conducted high-frequency (100 kHz) capacitance-voltage (C-V) measurements. Figure 2a depicts the bidirectional C-V characteristics for a 20 × 20 μm^2^ capacitor annealed at different temperatures. For samples with as-deposited La_2_O_3_ film, the physical thickness is about 5 nm and the EOT value extracted from C-V measurement was 1.01 nm. Significant changes at different annealing temperatures are observed. Figure 2b plots the normalized peak capacitance value and hysteresis shift as a function of annealing temperature. The capacitance value in general decreases for higher annealing temperatures (>400 °C). It should be mainly due to the growth of the interface silicate layer. The large hysteresis of the bi-direction voltage scan in the as-deposited sample indicates a large amount of oxide traps. The almost zero hysteresis of bi-directional C-V scan and the reduction in border trap bounce in the transition region of the 500 °C and 600 °C annealed samples should also be due to the formation of interfacial SiO_2_ which should have much better interface properties than La_2_O_3_ and the removal of oxygen vacancies in La_2_O_3_. There was a slight increase in the capacitance value for the sample annealed at 300 °C and 400 °C. This result can be explained by the increases in interface surface roughness. The amorphous silicate layer formed by the rather non-uniform oxygen diffusion can make the interface rougher and the increases of thickness should be not significant at low temperatures [8,9,10]. For low-temperature annealing, both TEM and C-V results indicate that the interface layer growth is negligible. However, because of local oxidation in some spots due to the oxygen diffusion from the electrode, the interface becomes rougher. The increase in capacitance value for 300 °C annealed sample agrees with the surface roughness effect as proposed by Zhao et al. [11]. This conjecture is further supported by the anomalously large gate leakage current and its variabilities to be reported at the end of this section.

### 3.2. Interface Roughness Effects

In studying the metal/semiconductor contact, an anomalously large Schottky current was reported. A fundamental calculation revealed that the electron cloud near the metal surface depends on the flatness of the metal [12,13]. A comprehensive calculation from first principles indicated that the fluctuations of the electric field, measured as δ*E*/*E*_S_, can be approximated by [11]:

(1) $$\frac{\delta E}{E_{S}}=\Delta^{2}\overset{k_{c}}{\underset{0}{\int }}\frac{{\left(l_{cor}k\right)}^{2}}{{\left(1+a\left(l_{cor}k\right)\right)}^{1+r}}dk$$

where *E_s_* denotes the electric field resulted from a smooth surface, δ*E* is the increased electric field due to surface roughness; *k* is related to surface wave vector, Δ the normalized roughness; *l_cor_* the normalized correlation length; *r* the roughness exponent which is a measure of the degree of surface irregularity; *a* is a proportional constant. The integral in (1) is the summation of the roughness spectrum approximated by the self-affine fractal model [11].

**Figure 3:**
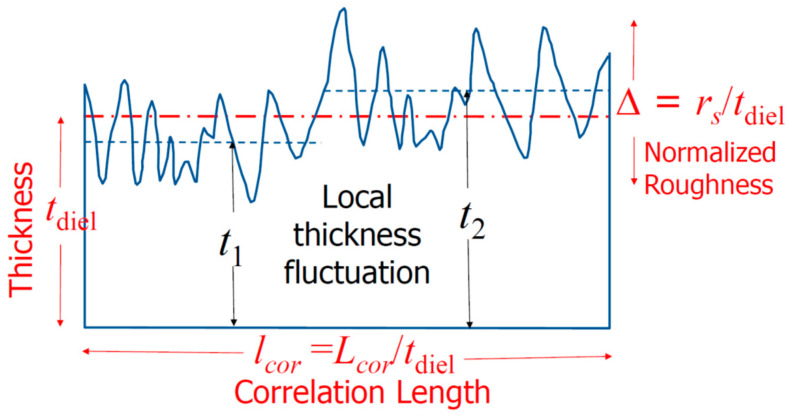
Definitions of key roughness parameters and illustration of local averaged thickness variation for small-sized devices as a function of the applied field.

It can be noted that both Δ and *l_cor_* are scaled with respect to the film thickness *t*_diel_. The roughness value (*r_s_*) can range from few angstroms to several nanometers and might be negligible if it is much smaller than the film thickness (*t*_diel_). However, when the film thickness is in a comparable range, the normalized roughness Δ (=*r_s_*/*t*_diel_) will be more significant. The second parameter governing the local field fluctuation is the normalized correlation length (*l_cor_*), which is also inversely proportional to the film thickness, i.e., *l_cor_* = *L_cor_*/*t*_diel_. This means that the thickness scaling would make both Δ and *l_cor_* and thus the electric field fluctuation larger. In short, both vertical and lateral scaling of the device to the nano-size, with device parameters close to the roughness value, will cause ever severe local electric field fluctuations. The definitions of some of the roughness parameters are illustrated in Figure 3.

A first-order approximation can be made as follows

(2) $$\frac{\delta E}{E_{S}}\approx \frac{r_{s}^{2}}{\alpha t_{diel}L_{cor}}$$

where α is an empirical constant governed by the irregularity of the roughness statistics.

Equation (2) reflects the major scaling issues arising from the surface roughness. For most cases, interface thickness and then surface roughness are not scalable parameters. For the same deposition technique, the *r_s_* value cannot be reduced for a thinner film. In contrast, when the film thickness approaching the atomic scale, the surface roughness would deteriorate. Thus, the electric field should be significantly enhanced as the *t_diel_* becomes thinner and *L_cor_* becomes smaller (in the case of smaller device size).

Instead of referring to a perfectly flat surface, we took the as-deposited film with roughness value, *r_s_*_0_, as a reference, and assuming the post-deposition processed film has a roughness value of *r_s_*_1_, then the change of electric field with reference to the as-deposited ones ($\delta E_{R0})\;$can be expressed as

(3) $$\frac{\delta E_{R1}}{\delta E_{R0}}=\frac{E_{R1}-E_{S}}{E_{R0}-E_{S}}={\left(\frac{r_{s1}}{r_{s0}}\right)}^{2}$$

Putting *r_s_*_0_ = 0.08 for as-deposited and *r_s_*_1_ = 0.12 for 300 °C annealed, it can be readily estimated that the actual local electric field in some 300 °C annealed sample can increase by 2.25 times with reference to the as-deposited ones.

For most of the current conduction mechanisms in insulators, such as Schottky emission, Fowler–Nordheim (FN) tunneling, and Poole–Frenkel (PF) emission, the current levels are exponential functions of the electric field [14]. Thus, the roughness-induced electric field fluctuations will entail a remarkable leakage current enhancement.

The impact of surface roughness on the electrical properties was realized decades ago in the metal-insulator-metal (MIM) structure as the lower interface (insulator on metal) is much rougher than the upper interface (metal on insulator). As a result, although the film is quite thick (>100 nm) and the size is quite large, obvious asymmetry forward and reverse currents were still observed [15,16]. This effect has now attracted more attention for the ultimate scale CMOS technology. By controlling the nitrogen concentration of the TiN gate electrode in a FinFET, it was found the workfunction of the gate electrode can vary from 4.71–4.82 eV by conduction C-V measurement [17]. This change should be mostly due to surface termination and nitrogen vacancies. However, the first principle calculation showed that the workfunction variation is smaller than the experimental values. In addition, it was also found that channel mobility is smaller for a high amount of nitrogen incorporation in the gate TiN which should have a low amount of nitrogen vacancies [17]. These inconsistencies may partially arise from the different surface roughness and are worth a detailed investigation. As the device size is being pushed closer to the atomic scale, the variability issues related to the surface roughness could be the main constraint for further device downsizing. In the next section, we shall present a detailed investigation of the gate current characteristics related to the processing condition and surface roughness.

### 3.3. Leakage Current Characteristics

In this section, we are going to demonstrate the interface and surface roughness effects with comprehensive measurements of the electrical characteristics of W/La_2_O_3_/Si capacitors. Noting that yet the gate dielectric thickness and device size are far larger than the targeted CMOS technology nodes, significant variability issues were still observed because of the instability of the high-k/Si interface. It manifests the importance of this issue in nano CMOS devices.

**Figure 4:**
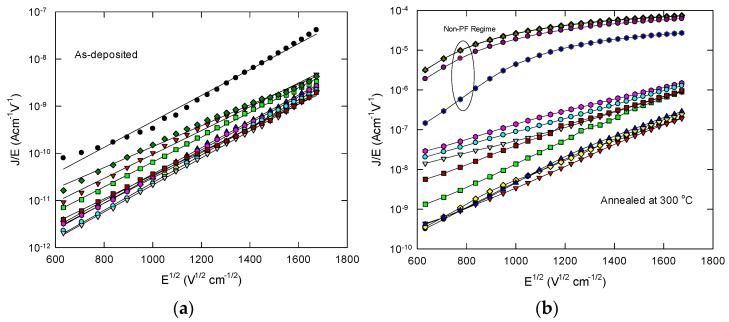
Poole–Frenkel plot of the current-voltage characteristic variability of W/La_2_O_3_/Si capacitors: (**a**) as-deposited; and (**b**) annealed at 300 °C for 30 min.

To study the device characteristic variabilities, we measured the leakage current characteristics of W/La_2_O_3_/Si capacitors at 20 different locations evenly on the same wafer for each of the as-deposited and 300 °C annealed samples. Capacitors near the edge region were avoided. It was suggested that leakage current is mainly due to Poole–Frenkel emission via dielectric traps at low electric fields [3]. Figure 4a,b shows the PF plot for the as-deposited sample and a sample with 300 °C thermal annealing for 30 min.

**Figure 5:**
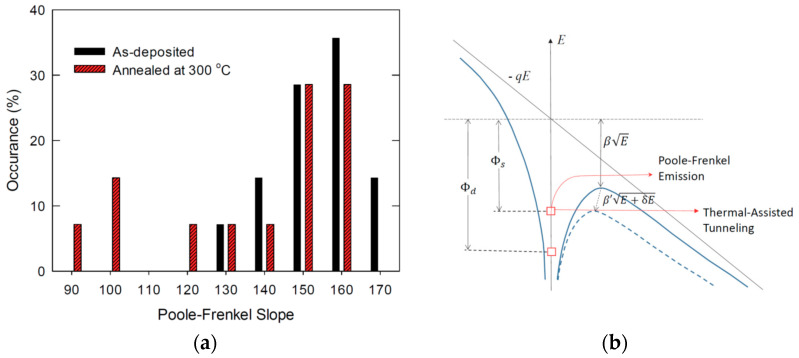
(**a**) Comparison of the distribution of Poole–Frenkel slope for as-deposited and 300 °C annealed samples. (**b**) Illustration of the possible mechanisms, electric field enhancement due to surface roughness (δ*E*), different conduction mechanisms involving shallow trap (Φ_s_), activation of deep trap (Φ_d_), change of dielectric constant (*β’*), leading to the different PF currents.

As shown in Figure 4a, the characteristics, in general, can be plotted quite well with the PF plot and most of the curves are essentially coincident or with rather small variations. It further confirms that the as-deposited La_2_O_3_ should have much smaller variations in both thickness, roughness, and trap energy level. For samples annealed at 300 °C (see Figure 4b), large variations of current-voltage characteristics, in both current level and slope, are observed at different locations. The magnitudes of leakage current characteristics were increased by one to two orders of magnitude. In addition, some curves depart greatly from the PF relationship with magnitude several orders higher. Figure 5 compares the PF slope distribution for as-deposited and 300 °C annealing samples. The majority of PF slope in a narrower range between 140 and 170 for the as-deposited sample. After 300 °C annealing, the PF slope extends to as low as 90. The large current and smaller PF slope can be partially attributed to the increased contributed contribution of Fowler–Nordheim (FN) conduction which will be discussed in detail in the latter part of this section. Other mechanisms can lead to the different behavior of PF conduction. Figure 5b illustrates the possible mechanisms giving rise to different PF slopes and current levels. Thermal annealing of high-k film usually results in the formation of a low-k interface layer. In some cases, the substrate silicon can even diffuse into the high-k layer and results in a smaller dielectric constant film and thus the smaller PF slopes (i.e., change of *β* to *β’* in Figure 5b). As confirmed with the TEM and XPS study [8], the 300 °C annealing is not enough to make this change. Hence, the smaller PF slope should not be due to this regime. The second mechanism can be the enhancement of the local electric field (i.e., *δE* in Figure 4b) due to the increased roughness. As estimated with (3), the local electric field can be enhanced by 2.25 times after 300 °C annealing. That makes the band bending/barrier of potential well lowering more significant (see the blue dashed curve on the right-hand side in Figure 5b). As a consequence, PF emission of shallow traps (with energy level denoted as Φs) is enhanced. In some cases, the thermally assisted tunneling can change into PF conduction because of the barrier lowering, and some deep traps with deep energy levels (Φd) can now contribute to the thermally assisted tunneling.

**Figure 6:**
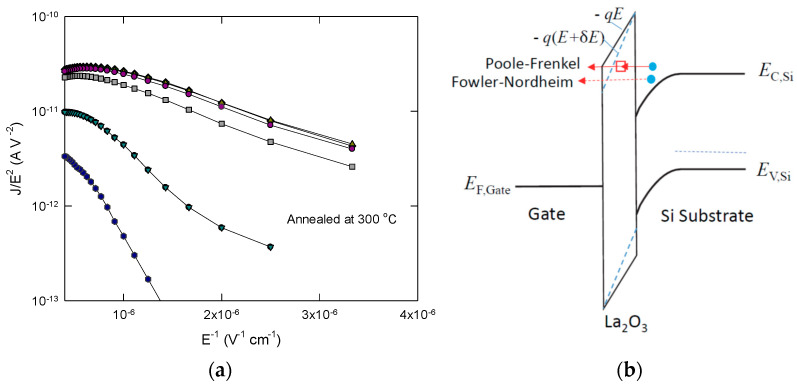
(**a**) Leakage currents at some locations of 300 °C annealed sample were several orders of magnitude larger than other locations and these characteristics can be plotted well with the Fowler-Nordheim relationship. The FN slope changes from 3.74–7.95 × 10^5^ V/cm. (**b**) A possible mechanism for the leakage current enhancement involving local electric field enhancement due to surface roughness (δ*E*). The barrier narrowing (blue dashed line) makes the FN tunneling possible (arrow with dotted line) and may convert the Poole–Frenkel conduction into FN tunneling (arrow with solid line).

In Figure 6, we re-plot the abnormally large leakage current with the Fowler–Nordheim relationship ($J_{FN}=AE^{2}\exp\left(-B/E\right)$) for the electric field in the range of 0.3 MV/cm to 1.1 MV/cm. In this field strength range, FN tunneling should not occur in our 4 nm thick La_2_O_3_. The anomalous large current could not be explained with any other conduction mechanism for this situation. As the interface La_2_O_3_/silicon has a small conduction band offset and as the effective mass of electrons in the conduction band is reduced, it was estimated that the direct tunneling limit is just slightly below 5 nm [2]. Although the thermal annealing at 300 °C should not cause significant interface growth, this process is still able to make both the top and bottom interfaces rougher. Follow the same rationale as proposed, we can attribute it to the high local electric field due to surface roughness. Thus, at some spots of high roughness, the local field strength should be able to make the FN tunneling occur. Figure 6 illustrates the narrowing of the triangular barrier due to the enhanced electric field. That makes the PF conduction in some cases evolve into FN tunneling and that is the reason for anomalously large leakage current for some locations. For these cases, the FN slope, $B=\frac{4\sqrt{2m_{e}^{*}}}{3q\hbar }\Phi^{3/2}$ [14], changes from 3.74 to 7.95 × 10^5^ V/cm because of the different roughness values.

The field strength modification due to surface roughness had been long realized [4,5]. It has always been ignored in the CMOS technological community as its effects were negligible because of the high uniformity of the thin film and excellent interface roughness control for the fabrication process [15,16]. Crystalline surfaces, such as metal films, are usually rougher because of the larger grain size and the uniformity limit of the employed evaporation or sputtering techniques. For this reason, the roughness effects were first observed in metal-insulator-metal (MIM) structures with larger sizes and much thicker films. Etching is another major cause for rough interfaces, due to the different etching rates of the target materials, non-uniform ion beams, and the energy distribution of ions in dry etching [17]. A typical dry-etched surface can have a roughness of several nanometers and a high value of roughness exponent [16]. Similar effects are expected to occur in other device structures and the processes involved in chemical or physical vapor deposition [18,19]. These processes have poor coverage and less film uniformity. The issue was not addressed as the film thickness and device size in most of the studies were quite large as compared to the roughness value. It is now a significant issue in the state-of-the-art nano CMOS technology [20,21]. It could also be a severe problem for the introduction of material with larger grain sizes [22] and 2D materials or in the devices based on 2D materials as the surface roughness value is even closer to the film thickness [23,24].

## 4. Conclusions

Although atomic-scale thin film deposition technique has been brought for the advanced fabrication process, the surface smoothness can readily be deteriorated because the reactions take place at the less thermal stability of high-k material and high-k interfaces. As the surface roughness is not a scalable parameter, it becomes more significant as the gate dielectric film is scaled down to the subnanometer EOT range. The surface roughness would have a significant impact on the device characteristics. In this work, we have revealed the increases of capacitance and leakage current variabilities of W/La_2_O_3_/Si MOS capacitors subjected to low-temperature thermal annealing. These observations can be explained consistently with the increase in surface roughness due to interface reaction in the La_2_O_3_ film. The formation of local nanocrystallites which are easily recognized [2,3] could also increase the surface roughness. Although this study only focuses on the I-V and C-V characteristics, it can be readily inferred that the thickness and local electric field fluctuations can lead to other parameter fluctuations such as surface potential, channel mobility, and low-frequency noise in the channel in addition to those effects on the gate. It is expected that similar consequences should occur in the other electronic devices when the surface roughness statistical parameters such as the relative roughness value, correlation length are comparable to the film thickness and device size.
